# Potential Role of Left Atrial Strain to Predict Atrial Fibrillation Recurrence after Catheter Ablation Therapy: A Clinical and Systematic Review

**DOI:** 10.3390/jcdd11070203

**Published:** 2024-06-30

**Authors:** Maria Barilli, Giulia Elena Mandoli, Nicolò Sisti, Aleksander Dokollari, Nicolò Ghionzoli, Hatem Soliman-Aboumarie, Flavio D’Ascenzi, Marta Focardi, Luna Cavigli, Maria Concetta Pastore, Matteo Cameli

**Affiliations:** 1 Department of Medical Biotechnologies, Division of Cardiology, University of Siena, Policlinico “Le Scotte”, 53100 Siena, Italyfocardim@unisi.it (M.F.); pastore2411@gmail.com (M.C.P.);; 2 Cardiology Unit, Electrophysiology Section, Gualdo Tadino Hospital, 06024 Gubbio, Italy; 3 Department of Cardiothoracic Surgery Research, Lankenau Institute for Medical Research, Wynnewood, PA 19096, USA; 4 Department of Anaesthetics and Cardiothoracic Critical Care, Harefield Hospital, Hill End Road, Uxbridge UB9 6JH, UK; hatem.soliman@gmail.com

**Keywords:** atrial fibrillation, catheter ablation, recurrence, advanced echocardiography, speckle tracking analysis, 3D echocardiography

## Abstract

Pulmonary vein isolation (PVI) with catheter ablation (CA) represents an effective therapy for atrial fibrillation (AF). Unfortunately, it is still not exempt from severe complications. The balance of risks and benefits should be assessed, and a patient-tailored approach is desirable. So far, several clinical and cardiac imaging parameters have been evaluated to investigate pre- and post-procedural features that could help clinicians in the selection of patients at high risk of a poor outcome after CA. This clinical and systematic review analyses the potential role of new LA parameters, such as LA reservoir strain, to predict AF recurrence after CA therapy. Notably, LA reservoir strain gains substantial clinical importance in patients with paroxysmal AF and when a low CHADS2-VASc score is retrieved. LA reservoir strain provides data concerning the risk of AF recurrence after PVI and, thus, in the management of long-term medical therapy in this patient’s group.

## 1. Atrial Fibrillation: Epidemiology and Therapeutic Strategy

Among cardiac tachyarrhythmias, atrial fibrillation (AF) stands out as the most prevalent worldwide, with current prevalence estimates in adults at approximately 2–4%, with 50 million cases recorded in 2020 [1,2]. Longer survival in older chronic patients, due to improved therapeutic management of comorbidities, allowed the incidence and prevalence of AF to reach the scale of a cardiovascular disease (CVD) epidemic of the 21st century [3]. AF is responsible for multiple cardiovascular and cerebrovascular complications, including heart failure and stroke, imposing a substantial burden on society, healthcare providers, patients, and global healthcare systems [4,5]. Therefore, a deeper understanding of the underlying mechanisms and the selection of patients for invasive treatment is mandatory [6].

Catheter ablation (CA) is a widely recognized technique to reduce AF recurrence [7]. It is an invasive but globally safe therapeutic alternative to antiarrhythmic drugs for the maintenance of sinus rhythm and for symptom improvement [8]. Several randomized controlled trials demonstrated a significant improvement of the quality of life within the ablation group vs standard medical therapy, indicating its usefulness for both paroxysmal and persistent AF [9,10,11,12].

In the latest American Heart Association (AHA)/European Society of Cardiology (ESC) Guidelines, CA by pulmonary vein isolation (PVI) is a recommended therapeutic option (Class I) following antiarrhythmic drug (AAD) failure, or patients’ AAD refusal, for paroxysmal AF or persistent AF, regardless of the presence of major risk factors for arrythmia recurrence. Moreover, it becomes a first line therapy in the setting of left ventricle dysfunction when AF tachycardia-induced cardiomyopathy is highly probable [1,2]. The efficacy of PVI in patients with AF is variable and differs between paroxysmal and persistent AF, with rates ranging from 60–70% to around 50%, respectively, with some differences considering early or late recurrences. Some 30–40% of patients undergoing CA require a second procedure [13,14,15]. An optimal selection is essential to mitigate unnecessary risks associated with CA procedures [16]. The identification and quantification of atrial stiffness and reduced compliance, secondary to wall fibrosis, are pivotal in this setting but challenging, mostly due to thin atrial walls. Second level echocardiography can better define tissue characterization when other imaging methods are not precise or feasible. In fact, the use of advanced techniques increases the accuracy for the analysis of myocardial deformation. The correct use of such methodologies can potentially improve AF in a patient’s clinical and therapeutical pathway and candidacy to CA with the main goal of reducing the burden of recurrence.

### Atrial Remodelling to Predict AF Recurrence

AF arises from left atrial scarring (LA), characterized by both electrical and structural changes. The latter are caused by increased fibrotic build-up, resulting in lesions which create re-entry currents of ectopic depolarization [2,17]. Pre-existing LA scarring and a low-voltage substrate are strong predictors of AF relapse after CA [18,19]. Through the characterization of LA remodelling prior to CA, the arrhythmogenic substrate can be identified, with a higher accuracy in predicting the effectiveness of CA itself. Among all available imaging techniques, some are particularly useful to assess atrial function, deformation, and remodelling. Cardiac magnetic resonance (CMR) and computed tomography (CT) can accurately describe atrial geometry and potentially wall fibrosis [20,21]. Nevertheless, in clinical settings, these imaging modalities remain relatively inaccessible in most centres and are expensive, requiring highly trained operators. On the contrary, transthoracic echocardiography (TTE) and tissue analysis is considered a gold standard thanks to its wide availability, ease-of-use and low-cost imaging modalities [22]. LA strain analysis by Speckle Tracking Echocardiography (STE) is a relatively recent technique that has been shown to accurately analyse myocardial deformation. LA strain application in the quantification of atrial remodelling has already been described for various cardiac conditions [23]. Its ability to avoid angle-dependency and enhance atrial fibrosis by deformation analysis, in addition to its strong correlation with invasive left-side filling pressures, makes it a powerful tool for these purposes [24,25,26].

## 2. Echocardiographic Predictors of AF Relapse

Every patient with a newly diagnosed AF requires a comprehensive clinical examination and TTE evaluation. The aim is the assessment of LA geometry, size and left ventricular (LV) diastolic and systolic functions. So far, several risk scores have been developed to predict patients at high risk of negative outcomes after pulmonary vein isolation (PVI), none demonstrating a strong capability of predicting recurrence. A promising evolution is awaited in the new machine learning era [27,28,29]. So far, the main clinical and echocardiographic predictive factors related to AF recurrence are gender, age, body mass index (BMI), AF pattern, heart failure, chronic kidney disease, LA size, and previous early AF recurrences [4].

### 2.1. Geometry and Size

An enlarged LA is recognized as an independent predictor of new-onset AF but also of a reduced success rate for PVI in terms of arrhythmia recurrence. In fact, atrial dilation contributes to structural remodelling, with consequent atrial fibrosis [30,31,32]. Among measures of LA size, left atrial diameter (LAD), in parasternal long axis view, has been widely used. Many investigators reported that a LAD > 45–50 mm is an independent predictor of AF recurrence, either measured by TTE or by transoesophageal echocardiography (TEE) [33,34,35]. In a meta-analysis with more than 3700 patients who underwent PVI, the recurrence rate was higher in patients with a weighted mean difference of LAD > 1.87 mm compared to a LAD measured in patients without recurrence (95% CI 1.26–2.48, *p* < 0.001) [36]. Pappone et al. applied TEE on 589 patients before PVI, reporting a LAD > 45 mm as a predictor of recurrence, either in patients with paroxysmal or persistent AF. However, LA dilation is often asymmetrical, and the LAD can underestimate true LA size [7,16]. The AFFIRM study showed that patients needing more than two cardioversions within the first year of antiarrhythmic medications often had a left atrial diameter over 45 mm, but with low sensitivity and specificity [37]. The ASE/EACVI recommendations classified the LA anteroposterior diameter as an unreliable index, giving higher importance to LA area or volume assessed by TTE [38].

Increased left atrial volume (LAV) or, better, indexed left atrial volume (LAVi), were linked to more frequent AF recurrences following CA, with an increased risk of recurrence of 3% for each additional millilitre of LAV/LAVi. These findings emerged from a meta-analysis including 3850 patients from 21 different studies assessed by TEE, where elevated LAV/LAVi was identified as an independent predictor for frequent AF recurrences after CA (OR—1.032, CI—1.012–1.052) [39]. According to the same meta-analysis, a LAVI > 34 mL/m^2^ had a sensitivity of 70% and a specificity of 91% in predicting AF recurrence [39]. Nonetheless, the assessment of LAV is constrained by low reproducibility due to the varying position, orientation of image planes, and interoperator variability. Similarly, studies focusing on LA area did not confirm a predictive accuracy [40]. 

In patients with diastolic dysfunction, an E/e′ ratio > 13–14 prior to CA relates to a higher risk of AF relapse. This was proved after both single and multiple procedures [41,42,43]. A relationship between a higher E/e′ ratio and low-voltage areas in the LA at electroanatomic mapping was described. This might indicate that a higher E/e′ is associated with an advanced atrial arrhythmogenic substrate, predisposing patients to a greater risk of relapse [22].

All the mentioned non-invasive parameters have demonstrated a variable capability for predicting the outcome of AF ablation but, unfortunately, none of them alone was accurate enough to enable an accurate prediction of AF relapse after CA [16]. Guidelines highlight that volumetric LA measurements should be cautiously considered because complex LA myocardial alterations linked to AF recurrence could not be detected by conventional atrial analyses of the atria [44]. In fact, many systematic reviews showed the benefits of risk prediction models for AF recurrence after CA, but a more robust evaluation is desirable with the application of a wider range of techniques [1,4,16,45,46].

### 2.2. Tissue Doppler Analysis: LA Strain

Tissue Doppler imaging (TDI) has been used to study LA wall structure via the analysis of atrial systolic strain to obtain more details on the mechanical characteristics and remodelling of its myocardium. Information regarding LA reservoir function and LA booster pump function can be given via the TDI-based analysis of LA peak systolic strain and late diastolic strain, respectively [47]. Schneider at al. evaluated the usefulness of TDI-based LA strain analysis in AF for the prediction of successful CA. The authors noted significantly higher values of LA strain and strain rates in patients that maintained sinus rhythm after CA. Additionally, an atrial septal systolic strain value of 20% before the procedure was predictive of maintained sinus rhythm after CA. However, these results were associated with relatively low sensitivity (57%) and specificity (56%) [48]. In a study involving 148 patients undergoing PVI, it was observed that LA strain and strain rate exhibited a significant increase from pre- to post-procedural follow-up. This could explain the possible role of LA systolic strain, measured at baseline, as a predictor of LA reverse remodelling [49]. The evaluation of LA strain by TDI, however, does not enable differentiation between active myocardial contraction and passive motion. Furthermore, it is prone to angulation error and limited by variable reproducibility [16].

Advanced echocardiography techniques like 2D and 3D Speckle Tracking Echocardiography (STE) are now validated and incorporated in the algorithm proposed by the EACVI consensus for the multimodality assessment of left heart filling pressures in HFpEF [24,50]. Their capability to define atrial reverse remodelling can strengthen the use of echocardiography in predicting successful AF ablation.

## 3. 2D Speckle Tracking Left Atrial Reservoir Strain after CA

### 3.1. LA Strain by 2D Speckle Tracking Echocardiography

The most recent EACVI/European Heart Rate Association (EHRA) Expert Consensus Document about multimodality imaging in AF recognizes LA strain, measured by STE, as a promising tool for the description of atrial myocardial mechanics [22]. Two-dimensional STE is a non-Doppler ultrasound-based method which allows a non-invasive quantification of myocardial deformation properties during the heart cycle. The process involves recognizing a speckle pattern within a predetermined region of interest (ROI) in the myocardium and assessing how the speckles move along the longitudinal plane, using statistical methods to pinpoint myocardial movements [26,51]. STE presents numerous benefits compared to traditional Doppler-derived indices: not only angle-independence but also lower reverberations, side lobes, and drop-out artifacts [45]. Moreover, it permits the analysis of deformation across the entire LA wall. STE facilitates regional function analysis and reduces intra- and interobserver variability. [17,52,53]. LA myocardial abnormalities can be identified by abnormal strain even when first level echocardiographic analyses are within the normal range. Moreover, the LA strain relationship with the degree of structural LA remodelling in patients with AF was validated [46,54,55,56]. These findings were also confirmed by Kuppahally et al., comparing the amount of fibrosis found by CMR with late Gadolinium enhancement and LA strain. LA fibrosis by CMR was significantly associated with mid-lateral strain and strain rate, regardless of LA volumetric and size assessments [57].

### 3.2. 2D-STE LA Reservoir Strain for Prediction of AF recurrence

**Figure 1:**
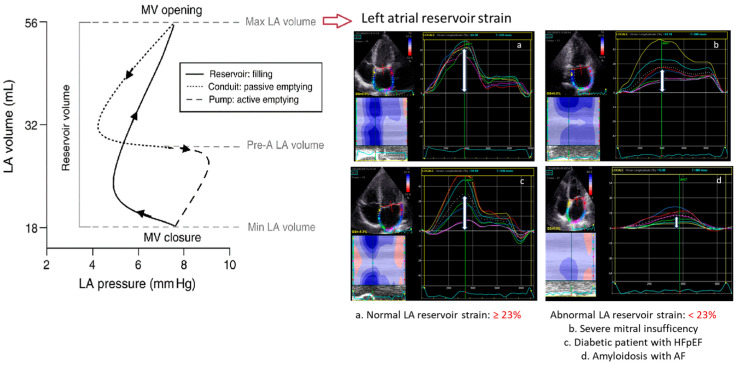
Limit of normality of 2D speckle tracking LA reservoir strain to assess LA function.

LA phases during each cardiac cycle are represented by (1) reservoir, (2) conduit, and (3) pump phase, which altogether are the expression of atrial compliance [58,59]. As shown by the recent Copenhagen City Heart Study, the reservoir function is assessed by the positive peak of the strain curve at the end of atrial filling, also described in the literature as peak atrial longitudinal strain. LA reservoir strain is the parameter with the higher sensitivity in identifying myocardial wall fibrosis (Figure 1) [60]. The pump (or contraction) phase is symbolized by the peak atrial contraction phase (PACS), while the difference between LA reservoir strain and PACS denotes the passive filling conduit phase (LACS) [58]. Baseline LA reservoir function, measured before CA, emerged as an independent predictor of LA reverse remodelling in both paroxysmal and persistent AF [45,61,62]. 

### 3.3. Clinical Relevance of LA Reservoir Strain to Predict AF after CA in Patients with Low CHA2DS2-VASc Scores and Paroxysmal AF

**Figure 2:**
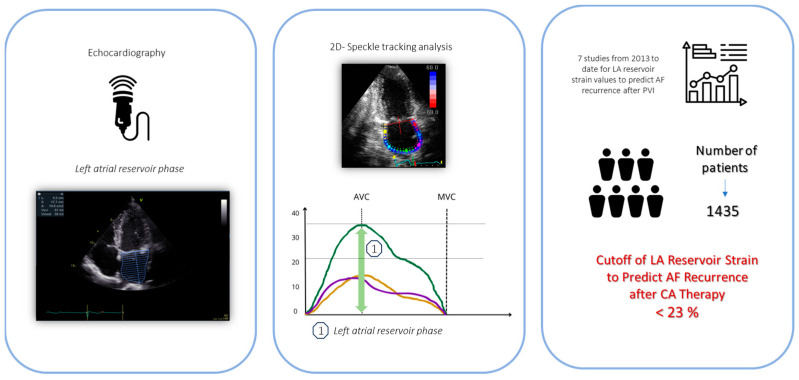
Usefulness and cutoff of LA reservoir strain to predict AF recurrence after CA therapy [55,56,61,66,67,68,69].

The clinical relevance of LA reservoir strain is underscored by its ability to serve as a non-invasive marker for atrial health. According to current large studies in healthy adult subjects, the lower limit of normality, or 2.5th percentile, for LA reservoir strain is 23% (Figure 1). This was proven by three important large studies, including a total of 3783 healthy subjects, that analysed the normal range of LA strain [63,64,65]. These studies showed similar results, with the lower limit of normality or 2.5th percentile for LA reservoir strain being 23% (see Figure 2). In agreement with these findings, several studies found also that a cut-off of LA reservoir strain < 23% was associated with a higher risk of AF recurrence following CA (see Table 1). Therefore, patients with LA reservoir strain below 23% may require closer monitoring and potentially more aggressive management post-ablation to mitigate the risk of AF recurrence. Morris et al. highlighted the potential role of LA reservoir strain in predicting AF recurrence after CA especially in populations with paroxysmal AF and low CHA₂DS₂-VASc scores (i.e., a score of 1 in women or a score of 0 in men), showing LA reservoir strain implications for a long-term therapeutic approach (e.g., anticoagulation) in this patient group [55]. For accurate assessment, it would be important to analyse LA reservoir strain in patients during sinus rhythm. This is particularly relevant in patients with paroxysmal AF, where intermittent episodes of AF are interspersed with periods of physiological sinus rhythm. In these patients, evaluating LA reservoir strain during sinus rhythm can provide a clearer picture of atrial function without the confounding effects of ongoing atrial fibrillation. Assessing LA reservoir strain in sinus rhythm helps in identifying subtle atrial dysfunctions that might predispose AF recurrence post-ablation.

The key point is that a significantly reduced LA reservoir strain before CA is suggestive of more severe fibrotic atrial remodelling, thus portending a higher possibility of failure or earlier recurrence after PVI. Additionally, the lack of improvement of LA reservoir strain after CA is an additional feature of a higher likelihood of new episodes of arrhythmia [56].

A relatively recent meta-analysis focused on the lack of data concerning the use of LA reservoir strain for CA selection. It included eight studies and 686 patients with the aim of identifying predictors of AF relapse after the procedure. Volumetric and geometric features and LV ejection fraction did not significantly vary among the two populations. On the contrary, a cut-off value of LA reservoir strain < 22.8% (range 18.8–30%, AUC 0.798 with *p* < 0.01) predicted AF recurrence, with a sensitivity of 78% and specificity of 75% [54]. Similarly, Yasuda et al. reported a reservoir LA strain value < 25.3% having an 81% sensitivity and a 72% specificity in predicting AF recurrence after the procedure [66]. In a comprehensive review of 10 studies, LA reservoir strain values were again compared to conventional parameters, such as LAD and LAVI, to predict AF recurrence. The analysis identified a threshold of 21.9%, with a higher risk of relapse below this threshold. LA dimensions (LAD and LAVi) were outperformed by LA reservoir strain in the prediction of recurrence, indicating a better insight when evaluating function over dimensions for recurrence risk prediction [46]. Hammerstingl et al. examined 103 patients with either paroxysmal or persistent AF, alongside 30 controls, over a follow-up period of 6 months. LA global strain parameters outperformed regional LA function analysis in predicting AF recurrences, with a sensitivity exceeding 85% and a specificity over 90% [62]. As discussed before, the role of the LA reservoir phase is a reflexion of LA wall fibrosis. It has high clinical relevance, feasibility, and reproducibility, especially when the amount of LA fibrosis is more likely to be low (namely, patients with paroxysmal AF when signs and symptoms have been detected < 48 h). It was also shown to be relevant when patients were in sinus rhythm, increasing the degree of accuracy in paroxysmal AF patients. The most recent study on this topic included 678 patients followed for 12 months after the procedure. LA deformation analysis by 2D STE revealed LA reservoir phase strain as an independent predictor of recurrence where OR = 1.04 per 1% of decrease (*p* = 0.015). They found an optimal cut-off range of <25.2 % (OR = 1.58, 95% CI [1.15; 2.15], *p* = 0.004) to significantly predict AF relapse [56].

STE atrial parameters can also play a role when the level of atrial remodelling is scarcely predictable, as in persistent AF patients. Concerning long standing AF, treatment and management remain challenging, but the overall duration of AF was proven as a negative predictive marker for the recurrence risk. Parwani et al. studied LA strain by 2D STE in 102 patients with persistent atrial fibrillation before CA. Patients with LA reservoir strain < 10% experienced a notably elevated recurrence rate (97.7%) throughout the entire follow-up period. Conversely, LA volumetric properties were similar between patients with and without AF recurrence, and there were no significant predictors in terms of AF duration or the type of CA procedure [67]. Another study included 100 patients with persistent AF treated with antiarrhythmic drugs for 3 months before the procedure. Failure of preprocedural sinus rhythm and LA reservoir strain were independently associated with high recurrence after the last procedure, with an LA reservoir strain cut-off ≤ 8.6%. These findings show how 2D STE can be useful to determine AF ablation candidates and also useful for patients where atrial degeneration has a higher probability to be severe and irreversible [68]. All recent studies on LA reservoir strain to predict AF recurrence after PVI are summarized in Table 1, with their graphical summary shown in Figure 2. 

## 4. Other Advanced Echocardiography Techniques for the Prediction of AF Recurrence: 3D Echocardiography and 3D STE

As previously described, LA volumes by 2D echocardiography were depicted to create standardization and classification criteria, to predict levels of atrial involvement in AF, and to define the probability of recurrence after ablation therapies. The prognostic value of 2D echocardiographic parameter definition in AF relapse risk needs, however, to be implemented by more accurate methods, as it is limited by geometric assumptions and scarce reproducibility due to variations in the position and orientation of imaging planes [22]. Advanced imaging modalities like computed tomography, magnetic resonance imaging, and 3D echocardiography have demonstrated higher accuracy when definition of atrial volumetric measurements is required, showing increased rates of arrhythmia recurrence in patients with larger LA volumes [21,70,71]. 

### 4.1. LA Function Analysis by Real-Time 3D Echocardiography

Among the mentioned imaging modalities, real-time 3D echocardiography (3DE) is a non-invasive advanced method showing an incremental prognostic value for the definition of atrial dilatation and structural remodelling [72]. It offers a precise quantification of LA volume and an automated evaluation of LA function. A study including 92 patients validated the use of 3DE for the direct measurement of LA volume compared to CMR images and 2DE-based analysis. The findings revealed a smaller number of patients with undetected atrial enlargement when analysed through 3D echocardiography compared to the CMR reference [73]. Marsan et al. studied the LA volumes and LA functions of 57 patients referred for CA, either affected by paroxysmal or persistent AF, and followed for 7.9 ± 2.7 months. Three months after the procedure, a successful CA was related to a decrease in LA volumes and a significant improvement in LA booster and reservoir functions. Conversely, individuals experiencing arrythmia recurrence displayed a tendency toward worse LA volumes and functions. Moreover, improved LA reservoir strain suggested authentic reverse remodelling linked to the lasting effectiveness of successful CA [74]. In a separate investigation involving 154 patients undergoing both 2D and 3D TTE and TEE before AF CA, LA reservoir function was assessed by expansion index difference. This parameter is measured with the formula [(Vmax3D2 Vmin3D)/Vmin3D × 100] on the average of three cardiac cycles (if sinus rhythm) or five cardiac cycles (if registered during AF). Using multivariate analysis, hypertension and the LA expansion index were the only independent predictors of successful therapy, serving as proxies for LA diastolic function. An LA expansion index > 103% was negatively associated with arrhythmia recurrence (OR 0.99, 95% CI 0.980–0.998, *p* < 0.019). An LA diameter > 51.5 mm and an LA maximal volume by 3D > 82.3 mL were also statistically related to unsuccessful procedures with high specificity (69–98%) but low sensitivity (10–16%) [75]. 

### 4.2. 3D Speckle Tracking Echocardiography

Three-dimensional LA speckle tracking analysis offers spatial insights into the whole cardiac chambers and precise measurements of the LA cavity. As opposed to 2D speckle tracking, LA function is assessed not only along the longitudinal but also along the circumferential axis [76,77,78]. So far, few studies have focused on 3D STE as a predictor of AF recurrence after CA, and the majority sampled patients with paroxysmal AF. Mochizuki et al. analysed the superiority of 3D STE over 2D STE in 42 patients with paroxysmal AF undergoing CA through the use of left atrial 3D global longitudinal (GLS), circumferential (GCS), and area strain (GAS) methods during LV systole and just before atrial contraction. Multivariate analysis indicated 3D GAS as an independent predictor of AF recurrence. The optimal cut-off value of pre-procedural 3D GAS was 28.9%, with a sensitivity of 75% and a specificity of 67% (HR 0.96; 95% CI: 0.91–0.9995, *p* = 0.048) for arrhythmia relapse. Moreover, 3D GAS proved to be a superior predictor compared to 2D LA strain or other established predictors [77]. More recently, a value > 5.89% for 3D left atrial pump strain (3D LAPS) was also reported as a predictor of AF relapse (HR = 1.40; 95% CI: 1.01–1.92, *p* = 0.040) and confirmed the superiority of 3D over 2D strain values as risk of recurrence predictors [79]. Kobayashi et al. applied 3D STE to determine novel parameters of LA mechanics through the analysis of electro-mechanical dispersion values. Research using 2D STE has already indicated an improved mechanical characteristic of the left atrium through the restoration of LA mechanical synchrony. A value of left atrial mechanical dispersion higher than 24 msec was identified as predictor of paroxysmal AF recurrence [80]. The main underlying assumption in these studies is that advanced LA fibrosis impedes a uniform relaxation of the LA, resulting in chamber dyssynchrony during the LA reservoir phase. The decrease in dispersed atrial regional deformation is reflected by enhanced standard deviation of the strain time to peak (TP-SD) of atrial segments, a parameter used to assess mechanical dispersion in patients with AF before and after cardioversion [81]. In 30 patients with paroxysmal AF TP-SD, GAS, and LA, volume exhibited significant improvement from pre- to post-pulmonary vein isolation. However, despite improvement after PVI, TP-SD remained significantly elevated, and the GAS was lower compared to controls (OR 1.21; 95% CI 1.04–1.49; *p* = 0.01) [76]. Therefore, TP-SD could represent a surrogate marker of LA fibrosis identifying atria with an advanced arrhythmic substrate. The results from all the described investigations seem to suggest 3D STE as an ally, together with 2D STE, for the quantification of LA dysfunction, considering the inhomogeneity of LA fibrosis through the chamber wall. The analysis of deformation across both longitudinal and circumferential axes using 3D makes it more accurate for atrial characterization. More studies are needed to analyse 2D STE and 3D STE for the prediction of AF CA success vs. failure in the short and long term, to implement their use in clinical practice, and to help clinicians in everyday practice. The main LA strain and 3DE parameters described in this review are summarized in Table 2. 

## 5. Practical Use and Limitations

LA reservoir function assessed with 2D STE and all the other mentioned advanced echocardiographic techniques can non-invasively quantify LA remodelling, helping the identification of patients with poorer outcomes following CA in terms of arrhythmia suppression. The performance and accuracy of such techniques before PVI are higher in paroxysmal AF patients and also when echocardiography is recorded in a sinus rhythm, making it more reproducible in patients where LA stunning is lower. According to our experience, the learning curve regarding 2D STE is fast, and a good level of analysis is easy to obtain even after a short learning period. LA reservoir strain and peak atrial contraction strain are both characterized by high feasibility and reproducibility. Both 2D STE and 3D STE need a good echocardiographic window (but it is also feasible in most sub-optimal windows); two and four apical chambers view images, recording with a stable electrocardiographic trace of three cardiac cycles (five cycles in patients with AF at the time of acquisition). Of course, accurate analysis, and consequently clinical significance, would be reduced if chamber walls are not rigorously recorded, including a complete visualization of the atrial endocardial layer. Usually, a dedicated echocardiographic section, with a partial foreshortening of the LV apex, is needed to guarantee a LA optimal view, and good training of the operators is mandatory to achieve this goal. If a previous limitation of LA strain was the lack of dedicated software for the analysis, automated online (on the echocardiographic machine) and offline software is now available for some vendors. In conclusion, these techniques are fast spreading nowadays, and their application is growing in many cardiological fields. The software is commonly available in many centres, also in peripheral hospitals. The availably of online software with a direct analysis of LA strain during the execution of echocardiographic exams without post-processing also reduces the time of execution. Three-dimensional echocardiography is now available in most centres too, with increasing applicability in daily practice both for LV and LA dimension and function estimation, and also for heart valve assessment. The technological software has been improved for more and more accurate performance.

In our opinion, in the future, all cardiologists and sonographers should be trained for the new second-level techniques, applying them during their daily practice in selected populations of patients, including for selecting AF patients for ablation therapy. Sensitivity and specificity of STE and 3D methods are higher than standard echocardiography measurements when tissue and volumes characterization are required, as in the case of the atrial chamber. This is why we apply them for a patient-tailored decisional pathway toward candidacy for CA in which we recognize LA reservoir strain as the more feasible and easy-to-be-measured parameter. We also consider LA reservoir strain as the more valuable tool to decide whether to select a patient for a second CA after AF relapse, to obtain higher sensitivity and improve our evaluation of electromechanical remodelling. However, we also recognize that more robust data and multicentric studies are still needed to improve the prediction of successful therapy and to increase sensitivity and PPV using echocardiographic techniques. Prognostic factors of CA success still need to be standardized. 

## 6. Conclusions

Given the growing evidence about the effectiveness of advanced echocardiography techniques in assessing the degree of atrial dysfunction and remodelling, their application becomes very useful in the setting of AF patients selected for CA therapy. LA reservoir strain, which estimates atrial compliance and reservoir function, has proven to be clinically significant, particularly in patients with paroxysmal AF and when recorded in sinus rhythm. For its accurate assessment, LA reservoir strain analysis during sinus rhythm is crucial, as it avoids the confounding effects of ongoing arrhythmia. The normal range of LA reservoir strain is also noteworthy, with the lower limit of normality (or 2.5th percentile) being 23%. Values below this threshold indicate impaired atrial function, which basically correlates with a higher risk of AF recurrence after CA therapy. Moreover, the measure of LA reservoir strain is crucial in patients with low CHA₂DS₂-VASc scores, providing functional and anatomical data on LA compliance and remodelling and aiding in choosing the best medical therapy and procedural planning. Combining LA geometrical and size features with LA reservoir strain analysis offers comprehensive functional and anatomical data on LA, paving the way for an accurate understanding of the pre-procedural risk of AF recurrence before pulmonary vein isolation.

## Figures and Tables

**Table 1:** Systematic review on the potential role of LA reservoir strain to predict AF recurrence after CA therapy. Inclusion criteria: sample size > 80 patients; follow-up > 12 months; CBI: cavotricuspid isthmus block; PVI: pulmonary vein isolation.

Study	Sample Size	AF Type	Catheter Ablation	Follow-Up (Months)	Cutoff of LA Reservoir Strain Predictor of AF Recurrence	OR or HR
Morris et al., 2013 [55]	84	Paroxysmal	PVI	19.2 ± 5.4	<18.8%	OR 6.8, 95% CI [21–21.4] *p* = 0.0011
Motoki et al., 2014 [61]	256	Paroxymal and Persistent	PVI	12	<23.2%	HR 0.939, 95% CI [0.916–0.962] *p* < 0.001
Yasuda et al., 2015 [66]	100	Paroxymal and Persistent	PVI	12	<25.27%	OR 0.81, 95% CI [0.73–0.89] *p* < 0.0001
Parwani et al., 2017 [67]	102	Persistent	PVI	15	<10%	HR 6.4, 95% CI [2.4–16.9] *p* < 0.001
Ma et al., 2017 [69]	115	Paroxysmal and Persistent	PVI	12	Paroxysmal <20.2 ± 3.4% Persistent <18.1 ± 3.4%	HR 0.79, 95% CI [0.67–0.96]; *p* = 0.01 HR 0.81, 95% CI [0.71–0.93]; *p* = 0.004
Hanaki et al., 2020 [68]	100	Persistent	PVI ± CIB	34 ± 16	≤8.6%	HR: 3.89, 95% CI [1.65–9.17], *p* = 0.002
Nielsen et al., 2022 [56]	678	Paroxysmal and Persistent	PVI ± CIB	12	<25.2%	OR = 1.58, 95% CI [1.01; 1.07], *p* = 0.004

**Table 2:** Suggested advanced echocardiographic predictors of AF recurrence. GASs: global area strain during LV systole; LA MD: left atrial mechanical dispersion; LAPS: left atrial pump strain.

LA Parameter	Cut-Off to Predict AF Recurrence	Graphical Representation	Description
LA reservoir strain (see Table 1) 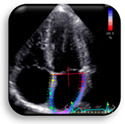	<23%	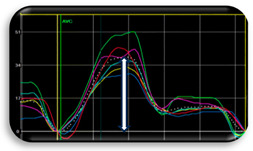	Peak atrial longitudinal strain quantifies atrial reservoir function, and Its decline suggests a higher level of atrial fibrosis.
LA MD [80]	>24 ms	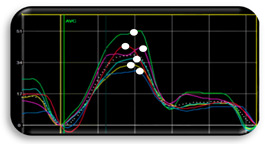	Left atrial mechanical dispersion represents inhomogeneous timing of contraction of atrial walls.
**3D Echocardiography**			
LA expansion index [75]	<103%		Left atrial expansion index (EI) is a parameter of LA reservoir function. Low EI represents a stiffer and less compliant atrium.
**3D Speckle tracking**			
3D–GASs [77]	<28.9%	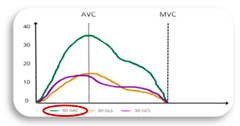	3D Global Area Strain quantifies left atrial area strain during LV systole (reservoir phase of the atrial cycle).
3D–LAPS [79]	<5.89%	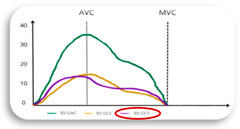	3D left atrial pump strain quantifies left atrial strain during the contraction phase of the atrial cycle.
